# Imaging CXCR4 Expression with Iodinated and Brominated Cyclam Derivatives

**DOI:** 10.1007/s11307-020-01480-1

**Published:** 2020-04-01

**Authors:** Hanwen Zhang, Masatomo Maeda, Masahiro Shindo, Myat Ko, Mayuresh Mane, Christian Grommes, Wolfgang Weber, Ronald Blasberg

**Affiliations:** 1 Department of Radiology, Memorial Sloan Kettering Cancer Center, New York, NY USA; 2 Department of Neurology, Memorial Sloan Kettering Cancer Center, New York, NY USA; 3 Department of Neurosurgery, Nozaki Tokushukai Hospital, Osaka, Japan; 4 Department of Nuclear Medicine, Technical University Munich, Munich, Germany; 5 Molecular Pharmacology & Chemistry Program, Memorial Sloan Kettering Cancer Center, Zuckerman Research Center (ZRC), Z-2060, 1275 York Avenue, New York, NY 10065 USA

**Keywords:** CXCR4, PET imaging, Radiolabeled cyclam derivatives

## Abstract

**Purpose:**

CXCR4 is one of several “chemokine” receptors expressed on malignant tumors (including GBM and PCNSL) and hematopoietic stem cells. Although ^68^Ga-pentixafor and ^68^Ga-NOTA-NFB have been shown to effectively image CXCR4 expression in myeloma and other systemic malignancies, imaging CXCR4 expression in brain tumors has been more limited due to the blood-brain barrier (BBB) and a considerable fraction of CXCR4 staining is intracellular.

**Methods:**

We synthesized 6 iodinated and brominated cyclam derivatives with high affinity (low nM range) for CXCR4, since structure-based estimates of lipophilicity suggested rapid transfer across the BBB and tumor cell membranes.

**Results:**

We tested 3 iodinated and 3 brominated cyclam derivatives in several CXCR4(+) and CXCR4(−) cell lines, with and without cold ligand blocking. To validate these novel radiolabeled cyclam derivatives for diagnostic CXCR4 imaging efficacy in brain tumors, we established appropriated murine models of intracranial GBM and PCNSL. Based on initial studies, ^131^I-HZ262 and ^76^Br-HZ270-1 were shown to be the most avidly accumulated radioligands. ^76^Br-HZ270-1 was selected for further study in the U87-CXCR4 and PCNSL #15 intracranial tumor models, because of its high uptake (9.5 ± 1.3 %ID/g, SD) and low non-specific uptake (1.6 ± 0.7 %ID/g, SD) in the s.c. U87-CXCR4 tumor models. However, imaging CXCR4 expression in intracranial U87-CXCR4 and PCNSL #15 tumors with ^76^Br-HZ270-1 was unsuccessful, following either i.v. or spinal-CSF injection.

**Conclusions:**

Imaging CXCR4 expression with halogenated cyclam derivatives was successful in s.c. located tumors, but not in CNS located tumors. This was largely due to the following: (i) the hydrophilicity of the radiolabeled analogues—as reflected in the “measured” radiotracer distribution (LogD) in octanol/PBS—which stands in contrast to the structure-based estimate of LogP, which was the rationale for initiating the study and (ii) the presence of a modest BTB in intracranial U87-CXCR4 gliomas and an intact BBB/BTB in the intracranial PCNSL animal model.

**Electronic supplementary material:**

The online version of this article (10.1007/s11307-020-01480-1) contains supplementary material, which is available to authorized users.

## Introduction

CXCR4 is a cell surface transmembrane G protein receptor; it is one of several “chemokine” receptors expressed on malignant tumors (including glioblastoma-GBM and primary CNS lymphoma-PCNSL) and on hematopoietic stem cells [1]. CXCR4 is highly expressed on aggressive/malignant tumor cells and plays a critical role in the homing of cancer cells to distant sites following binding to its ligand CXCL12. Chemokines (small proinflammatory molecules that bind to specific G protein-coupled receptors) are major regulators of cell trafficking and adhesion. The chemokine CXCL12 (stromal cell-derived factor-1 (SDF-1)) binds primarily to CXC receptor 4 (CXCR4), establishing CXCL12–CXCR4 signaling pathway and are involved in the cross-talk between cancer cells, stromal cells, T cells, and their microenvironment, including the regulation and direction of T cell migration (chemotaxis), proliferation, and differentiation of immature progenitor-stem cells [2].

High levels of CXCR4/CXCL12 expression in PCNSL [3–5] and in glioblastomas [6–8] have been shown previously. Activation of the CXCR4/CXCL12 pathway is considered to be a major contributor to tumor invasion of surrounding tissues [8, 9]. In GBMs, CXCR4 expression correlates with poorer patient prognosis [9] and with treatment response to AMD3100 (a clinically approved CXCR4 inhibitor) [10–12]. In PCNSL, there is little or no literature describing the association of high CXCR4 expression and prognosis.

Several inhibitors of the CXCL12–CXCR4 signaling pathway have been developed. Bicyclams were first synthesized in 1987 to assess the redox chemistry of dimetallic coordination compounds [13]. Serendipitously, bicyclams were discovered to have a potential use in the treatment of HIV, due to its role in blocking of CXCR4—a co-receptor for CD4+ T cells, in certain strains of HIV. AMD3100 (Plerixafor) was synthesized in 1994 and inhibited HIV infection in the 1–10 nM range [14]. AMD3100 is also approved for stem cell mobilization prior to autologous stem cell transplantation in patients with multiple myeloma and lymphoma. CXCR4 targeting with AMD3100 was also investigated as a therapy for leukemia and various solid tumors. In 2010, the feasibility of using radiolabeled [^64^Cu]AMD3100 to image CXCR4 expressing tumors was shown in animal models [15] and several other AMD3100 metal chelates, including ^99m^Tc and ^67^Ga, were subsequently studied preclinically [16–18]. Most experience with CXCR4 imaging, however, exists for a different CXCR4 binding ligand, namely, the cyclic pentapeptide [^68^Ga]Pentixafor, an analogue of CXCL12/SDF-1. Using this imaging agent, various CXCR4 expressing tumors were imaged successfully in patients [17, 19, 20]. Furthermore, a similar pentapeptide, labeled with the beta-emitter Lutetium (^177^Lu), has shown promise for targeted radiotherapy of CXCR4 expressing malignancies in early clinical studies.

However, imaging CXCR4 expression in brain tumors with ^68^Ga-pentixafor and ^68^Ga-NOTA-NFB [21, 22] has been less successful. [^68^Ga]Pentixafor was shown to have variable uptake characteristics in gliomas, the tumor SUVs were less than those of ^18^F-FET (although brain background was very low), and there was an inconsistent relationship between [^68^Ga]Pentixafor uptake and CXCR4 immunohistochemistry on resected tumor tissue [21]. There are at least two potential explanations for this limitation: (i) the blood-brain barrier (BBB) may restrict access of radiolabeled peptides to CXCR4-expressing tumor cells and (ii) a considerable fraction of CXCR4 staining in gliomas is intracellular and intracellular CXCR4 may not be accessible to the peptides. Issues related to the blood-brain barrier (BBB) and blood-tumor barrier (BTB) could be problematic. These issues have not been fully addressed.

We previously synthesized ^18^F-labeled pyrimidine-pyridine amine (LogD at 1-octanol/PBS = 1.4) with aim to pass through BBB and BTB. Unfortunately, our leading ligand, ^18^F-3**,** showed low affinity to CXCR4 [23]. Considering that positive clinical results have been obtained with FDA-approved Plerixafor and AMD3465 and that a ^64^Cu-chelate of both Plerixafor and AMD3465 did show promising accumulation in CXR4-positive xenografts [15, 24, 25], we considered that modifications to the cyclam and pyridine may have resulted in a significant loss of affinity to CXCR4. Therefore, we considered that (radio)halogenation on the phenyl moiety of either Plerixafor or AMD3465 would cause a minimum effect on its affinity to CXCR4, which was the rationale for current studies. Furthermore, introducing radioiodine or radiobromine on the phenyl moiety might result in more effective transfer across the blood-brain barrier and therefore enable better imaging of CXCR4-expressing brain tumors and that the halogenated analogs could serve both a CXCR4 imaging and targeted radiotherapy function. To investigate this hypothesis, we designed and synthesized six small molecule CXCR4 inhibitors as potential anti-CXCR4 theranostic agents. The radiolabeled analogs were evaluated using *in vitro* cell binding assays and *in vivo* PET imaging of appropriated murine models with s.c. and intracranial tumors.

## Materials and Methods

### Synthesis of Halogenated Small Molecule Inhibitors of CXCR4

Tri-tert-butyl-1,4,8,11-tetraazacyclotetradecance-1,4,8-tricarbonate (73 mg, 0.146 mmol pre-dissolved in 1 ml of THF) was added into 1,4-bis(bromomethyl)-2-bromobenzene (50 mg, 0.146 mmol) in 1 ml of THF. After mixing well, diisopropylethylamine (0.161 mmol in 0.5 ml THF) was added dropwise, and the reaction mixture was stirring at room temperature for overnight. After removal of solvents and purification on silical chromatography column, the mixture of HZ267-1 and HZ267-2 was obtained for further reaction with 0.73 mmol of 2-picolylamine at room temperature in THF for 1 day. After purification on silical chromatography column, 1 h deprotection with 95 % TFA, and removal of trifluoroacetic acid (TFA) with rotary evaporation, the pure HZ270-1 and HZ270-2 were obtained from the collected fraction of HPLC purification (column: Waters C18, 19 mm × 250 mm, 5 μm, 100 Å; UV detector 280 nm; flow rate 15 ml/min; mobile phase 0.1 % TFA in water and acetonitrile (MeCN)); gradient 0–1 min, 2–10 % MeCN, 1–11 min, 10–35 % MeCN, 11–11.5 min, 35–95 % MeCN, 14.5 min, 95 % MeCN, 15 min, 2 % MeCN) and characterized with UPLC-MS system (flow rate 0.3 ml/min; mobile phase 0.1 % TFA in water and MeCN; gradient 0–5.5 min, 2–15 % MeCN, 6.0–7.5 min, 95 % MeCN, 8 min, 2 % MeCN). For synthesis of HZ261, three equivalents of 1,4,8,11-tetraazacyclotetradecane were reacted with 1,4-bis(bromomethyl)-2-bromobenzene for 1 day at room temperature. Using a similar procedure of synthesizing brominated ligands, 1,4-bis(bromomethyl)-2-iodobenzene was utilized instead of 1,4-bis(bromomethyl)-2-bromobenzene for preparation of all iodinated derivatives (HZ262, HZ265, HZ271-1 and HZ271-2). It is worth noting that efficient separation of the two isomers (*e.g.*, HZ270-1 *vs* HZ270-2; HZ271-1 *vs* HZ271-2) was achieved due to a 1-min difference retention time between the two isomers. A relatively small amount of mass (< 5 mg) was loaded on the preparative HPLC system for each purification run.

### Competitive Binding Assays

In order to determine the affinity of the halogenated cyclam derivatives, competitive binding studies were performed with U87-CXCR4 cells using ^67^Ga-Pentixafor (CXCR4 receptor ligand) [26]. Briefly, triplicate samples containing 0.5 × 10^6^ cells pre-seeded in 6-well plate, about 1.8 kBq of ^67^Ga-Pentixafor (4.0 nM) and 0.001–1000 nM of the tested ligands (total volume 1.0 ml) were incubated at 37 °C for 2 h. After incubation, the cells were washed with 2 × 1 ml of ice-cold tris-buffered saline (pH 7.4) and then collected for measurement with a WIZARD 1480 gamma counter (PerkinElmer, Waltham, MA). The IC_50_ values were estimated using a least squares fitting routine (GraphPad Prism 6, San Diego, CA, USA).

### Radiosynthesis of Halogenated Small Molecule Inhibitors of CXCR4

**Fig. 1:**
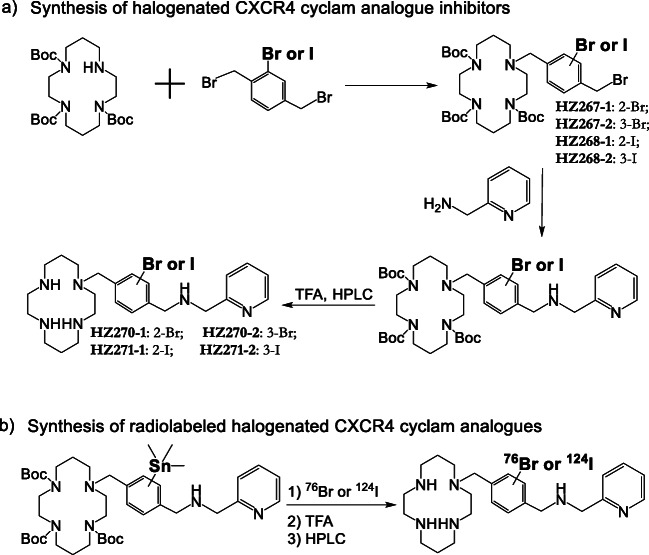
Synthetic scheme of halogenated CXCR4 inhibitors: cyclam derivatives. **a** Synthesis of halogenated CXCR4 cyclam analogue inhibitors. **b** Synthesis of radiolabeled halogenated CXCR4 cyclam analogue inhibitors.

Custom synthesis was utilized for preparation of all three trimethylstannane precursors of the cyclam derivatives (Fig. 1b). The precursor (2.0 mg per ml of water) was aliquoted to 10 μg per vial. ^76^Br (or ^77^Br) (185 MBq 5–20 μl of 0.1 M NaOH solution) was added into the precursor vial, followed by 10 μl of acetic acid, and 5 μl of chloramine-T (2.0 mg/ml acetic acid) solution. The resultant mixture was incubated for 10 min at room temperature, and then 500 μl of trifluoroacetic acid was added into the resultant mixture for 30 min incubation. After the solvents in the reaction vial were removed with nitrogen flow under 50 °C, the residual in the reaction vial was re-dissolved with 200 μl water and loaded to a radiodetector-integrated HPLC system for purification (column: Luna C8 [17], 100 Å, 4.6 × 250 mm; flow rate 1.2 ml/min; mobile phase 0.1 % trifluoroacetic acid in water and MeCN; gradient 0–10 min, 10–30 % MeCN; 11–14 min, 95 % MeCN; 15 min, 10 % MeCN). The collected fraction was evaporated and re-dissolved with PBS to generate the final product ^76^Br-HZ270-1, which was further confirmed by co-injection of the corresponding standard. The same precursor was also utilized for the preparation of ^131^I-HZ271-1. The same protocol of ^76^Br-HZ270-1 radiosynthesis was used for all other *Br/*I-labeled ligands that are listed in the Table 1.

**Table 1:** Halogenated small molecule inhibitors of CXCR4

	Structure	Partition coefficient estimated LogP^§^	Partition coefficient measured LogD*	IC_50_ affinity (nM)^†^
AMD3100		− 0.41	NA	6.30 ± 2.62
HZ261		0.42	− 1.48 ± 0.21	9.47 ± 2.87
HZ262		0.95	− 1.95 ± 0.06	15.9 ± 0.4
AMD3465		1.34	NA	15.0 ± 5.8
HZ270-1		2.17	− 0.72 ± 0.03	6.73 ± 0.72
HZ271-1		2.69	0.11 ± 0.02	7.27 ± 0.38
HZ270-2		2.17	− 0.83 ± 0.03	18.0 ± 7.0
HZ271-2		2.69	0.31 ± 0.09	32.1 ± 13.2
HZ265		4.44	ND	> 5000

*ND* not done, *NA* not applied

^§^Structure-based estimates of lipophilicity (LogP estimates) of the halogenated cyclam derivatives, were calculated using Chemdraw software (PerkinElmer, Professional v16)

*Measured partition coefficient studies in 1-Octonal/PBS (volume ratio 1/1) using carrier-free radiohalogenated ligands

^†^Competitive binding affinities were measured in triplicate with U87-CXCR4 cells and 67Ga-Pentixafor

### LogD Determination

The radiotracer distribution (LogD) was determined by partitioning the corresponding radioligand between 1-octanol and phosphate buffered saline (PBS, pH 7.4) (v/v = 1/1) as previously described [26]. The 1-octanol/PBS mixture was pre-equilibrated for at least 24 h at room temperature. The LogD values were calculated using the formula: logD = log_10_ (counts in 1-octanol layer / counts in PBS layer).

### Uptake of Radiohalogenated Cyclam Derivatives in Glioma and Lung Cancer Cells

Glioma (U87-CXCR4 and U87-WT) and lung cancer (H82 and H69) cell lines with different level of CXCR4 expression were utilized for cell uptakes of all synthesized six radiotracers. Each cell line (0.25 × 10^6^ per well) was seeded into 6-well plates, and on the next day, triplicate samples containing approximately 1.85 kBq of *Br or *I-labeled ligand were added and kept incubation at 37 °C for 2.0 h. After washing twice with PBS (4 °C) on the ice-bed, the cells were harvested for measurement with the gamma counter. Non-specific uptake was determined by co-incubating with 100 μM of AMD3100 (final concentration).

### Tumor Cells and Animal Tumor Models

A number of human and murine cell lines and PCNSL animal models were evaluated for CXCR4 expression (Table 2). CXCR4-transduced U87 glioma cells (U87-CXCR4) were obtained from the NIH AIDS Reagent Program (cat. no. 4036) [27]; U87 wild-type, Gl261, CT-2A, and ALTS1C1 glioma cells were obtained from the National Institute of Health, USA [28]; Dr. Thomas Seyfried (Boston College, Boston, MA) [29]; and the Bioresource Collection and Research Center(Taiwan) [30], respectively. H69 and H82 lung cancer cell lines were obtained from American Type Culture Collection (ATCC). All cell lines and intracranial tumors were assessed for CXCR4 expression by immunohistochemistry (IHC) and western blot. CXCR4 IHC was processed by the MSK core facility. Based on the IHC and western blot analyses, “high” and “low” CXCR4-expressing cell lines were selected for further *in vitro* uptake studies, comparing 6 radiolabeled cyclam derivatives. These studies included 2 human (U87-CXCR4, U87 wild type) glioma cell lines and 2 human lung cancer cell lines (H69, H82).

**Table 2:** Cell lines and orthotopic tumors

Tumor	Origin	Host	CXCR4 Cells†	
PCNSL				
PCNSL-5	Human	Nude		O/+
PCNSL-6	Human	Nude		+
PCNSL-10	Human	Nude		0
PCNSL-11	Human	Nude		+++
PCNSL-12	Human	Nude		0/+
PCNSL-14	Human	Nude		++++
PCNSL-15	Human	Nude		++++
PCNSL-16	Human	Nude		+++
Glioma				
U87	Human	Nude	0	0
U87-CXCR4	Human	Nude	++++	++++
GL261^#^	Murine	C57BL/6	0/+	+
CT-2A†	Murine	C57BL/6	0	+
ALTS1C1	Murine	C57BL/6	++	++
Lung cancer				
H69	Human	Nude	+++	
H82	Human	Nude	+	

All animal experiments were approved by the Institutional Animal Care and Utilization Committee of MSKCC. Eight human PCNSL xenograft models established at MSK (CG) were available for study. Female Athymic Nude Foxn (6–8 weeks old; ENVIGO) was used for subcutaneous implantation. Cancer cells, including U87-CXCR4, U87-WT, H69, and H82 (5 × 10^6^ cells suspended in 200 ml of cell culture medium/Matrigel [BD Bioscience] [v/v 5 1/1]), were inoculated to the shoulder/flank area of the animal. Twenty to 30 days after the inoculation, imaging and tissue sampling were performed when the tumor sizes were between 100 and 350 mm^3^. For orthotopic models, U87-CXCR4 cells (1 × 10^5^ cells suspended in 10 μl of cell culture medium) in 30-gauge needle were injected into the right frontal cortex (stereotactic coordinates: bregma + 1.7 mm (anterior), lateral − 0.5 mm (right), and at a depth of 2.5 mm). Individuals performing implantation and stereotactic injections were blinded to the experimental design.

### Magnetic Resonance Imaging

MRI was acquired prior to PET imaging. Mice were imaged on a 4.7-Tesla Biospec scanner with a 12-cm gradient coil, operating at 200 MHz (400 mT/m ID; Bruker Biospin MRI GmbH). Mice were positioned in the scanner by acquiring T2-weighted scout images 6 along 3 orthogonal orientations. T2-weighted mouse cranial images (slice thickness of 0.8 mm and a field of view of 30 × 34 mm with a spatial resolution of 117 × 133 μm) were acquired using fast spin-echo sequences.

### Small Animal PET or PET/CT

Animals were administrated 3.7–11.1 MBq of ^76^Br- or ^124^I-labeled ligands *via* tail vein or intraspinal injection under 2 % isoflurane anesthesia. At 1 h and 24 h after injection, PET/CT imaging (or PET imaging) was performed with an Inveon PET/CT system. The tumors were centered in the field of view, and the PET acquisition time was 10 min (or longer if needed, until 50 million events are recorded). The image data were normalized to correct for non-uniformity of response of the PET camera, dead-time count losses, positron branching ratio, and physical decay to the time of injection, but no attenuation, scatter, or partial-volume averaging correction was applied. The calibration factor of both PET scanner was measured with a mouse-size phantom composed of a cylinder uniformly filled with an aqueous solution of ^18^F of known activity concentration. The acquired PET/CT (or PET) images were analyzed using Inveon Research Workplace v4.2 software (or ASIPro software). Region of interest (ROI) analysis of the acquired images was performed using ASIPro software (Siemens, Malvern, PA, USA), and the observed maximum pixel value (%ID/cc) was utilized to diminish partial volume effects of tumor. The sequential radioactivity measurements (% injected dose/cc) were plotted *vs.* time of acquisition.

After the last PET imaging time point, the animals were euthanized for tissue dissection. The organs of interest were collected, rinsed of excess blood, blotted, weighed, and counted with a WIZARD 1480 gamma counter (PerkinElmer, Waltham, MA). The total injected radioactivity per animal was determined from the measured radioactivity in an aliquot of the injectate. Data were expressed as percent of injected dose per gram of tissue (%ID/g).

### Autoradiography and Immunohistochemistry

Following PET imaging or biodistribution studies, tumors were excised, embedded in optimal-cutting-temperature mounting medium (OCT, Sakura Finetek), and frozen on dry ice prior to cutting a series of 10 μm frozen sections. Digital autoradiography was performed on a phosphor imaging plate (Fujifilm BAS-MS2325; Fuji Photo Film) at − 20 °C. Phosphor imaging plates were read at a pixel resolution of 25 μm with a Typhoon 7000 IP plate reader (GE Healthcare). Following autoradiography imaging, the same section was stained with H&E and whole-mount bright field images acquired in a similar manner.

### Statistical Analysis

Data calculated using Microsoft Excel are expressed as mean ± SD. Student’s unpaired *t* test (GraphPad Prism 7, San Diego, CA, USA) was used to determine statistical significance at the 95 % confidence level. Differences with *p* values < 0.05 were considered to be statistically significant.

## Results

### Design and Synthesis of all Halogenated Small Molecules of CXCR4 Inhibitors

Based on the structure of Plerixafor (AMD3100) and AMD3465, there are two positions on the phenyl moiety of AMD3465 and one position on Plerixafor suitable for halgenation. Since iodination or bromination at same position may cause different *in vitro* and *in vivo* behavior, six different iodinated/brominated derivatives were designed. The structural detail is shown in Table 1. Using 1,4-bis(bromomethyl)-2-**x**benzene (**x** = bromo or iodo) as the starting material, all brominated/iodinated CXCR4 inhibitors were synthesized in 2–3 steps (Fig. 1a). After purification on a preparative HPLC system and lyophilization, milligram amounts of all halogenated ligands were obtained with a yield of 20 to 50 %, with the purity of greater than 98 % (these amounts were sufficient for *in vitro* binding assays.

### Competitive Binding Assays

Competitive binding assays were performed using U87-CXCR4 cells (expressing high levels of CXCR4) and ^67^Ga-Pentixafor [31, 32] as a radioligand. Representative competition curves are shown in Figure S-1. Compared to non-halogenated CXCR4 inhibitors (AMD3100 (Plerixafor, FDA-approved drug) and AMD3465), all newly synthesized compounds had similar IC_50_ values, in the low nanomolar range (Table 1), except HZ265, which had no affinity to CXCR4. This observation indicated that the cyclam group was most sensitive to maintain high affinity to CXCR4, and that (radio)halogenation on the phenyl moiety was a potentially promising approach to introduce radiobromine (^76^Br/^77^Br) or radioiodine (^123^I/^124^I/^131^I) for targeted PET/SPECT imaging or radionuclide-based targeted-treatment of CXCR4-positive cancer. It is worthy to note that bromination on the phenyl moiety had less influence on binding than iodination in the same position. In addition, halogenation at the 2nd position of phenyl moiety resulted in slightly lower IC_50_ values than halogenation at the 3rd position (HZ270-1 *vs* HZ270-2; HZ271-1 *vs* HZ271-2).

### Radiosynthesis of All Halogenated Small Molecules of CXCR4 Inhibitors

The trimethylstannane precursors (Fig. 1b) were designed to introduce both radiobromine and radioiodine with high yield at the selected sites on the phenyl moiety. All three precursors were synthesized successfully by our custom-service vendor and characterized with LC-MS, NMR, and high-resolution mass spectrum (Supplemental Figure S-2). Radiolabeling with ^124^I/^131^I and ^76^Br/^77^Br was performed under mild conditions to produce 3 radioiodinated and 3 radiobrominated CXCR4 inhibitors (Fig. 1b). After purification with a radiodetector-integrated HPLC system, all carrier-free radiotracers were obtained in two steps with an overall radiochemical yield of 30–50 %. The purity of the final 6 radiotracers was greater than 98 % (analyzed by HPLC), and the radioligand peak observed in HPLC chromatography was further confirmed by co-injection of the corresponding cold compound.

### LogD Determination

The structure-based estimates of lipophilicity (LogD estimates) of the halogenated cyclam derivatives were calculated using Chemdraw software (PerkinElmer, Professional v16). Subsequent measurements of the 1-octanol/PBS partition coefficient (LogD) of the corresponding radiolabeled compounds gave more hydrophilic values (Table 1). The reason for such difference between prediction value and experimental data is the prediction value is based on the atomic characteristic without consideration of protonation at PBS. Among the new radiolabeled ligands, the experimental data indicated that the bicyclam derivatives from Plerixafor were more hydrophilic than that of monocyclam derivatives with more protonated amines from AMD3465. In addition, bromination on the benzyl group caused more hydrophilicity than that of iodination in the same position (HZ270-1 *vs* HZ271-1; HZ270-2 *vs* HZ271-2).

### Uptake of Radiohalogenated Cyclam Derivatives in Glioma and Lung Cancer Cells

**Fig. 2:**
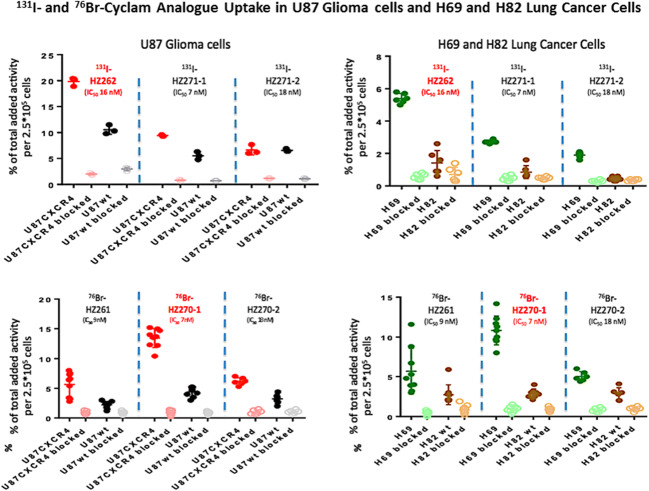
^131^I- and ^76^Br-Cyclam uptake in U87 Glioma cells and H69 and H82 lung cancer cells. The uptake of 6 cyclam derivatives; (^131^I- and ^76^Br-labeled) *was studied in* U87 CXCR4-expressing and wild-type glioma cells (left panels) and in H69 and H82 lung cancer cells (right panels) were studied. Uptake of ^131^I-labeled HZ262, HZ271-1 and HZ271-2 is shown in the upper panels; uptake of ^76^Br-labeled HZ262, HZ270-1 and HZ270-2 is shown in the lower panels. Non-specific uptake was determined by blocking specific uptake with 100 μM of AMD3100. Individual experiments are plotted.

The uptake of 6 different radiolabeled cyclam derivatives was studied in 4 glioma and lung cancer cell lines (Fig. 2). The cell lines expressed both “high” (U87-CXCR4 and H69) and “low” (U87-WT and H82) levels of CXCR4, which were confirmed with IHC staining of CXCR4. All 6 radiolabeled cyclam derivatives showed higher accumulation in both U87-CXCR4 and H69 cell lines than that in U87-WT and H82 cell lines, and radiotracer uptake was blocked to background levels in all cell lines by co-incubation of 100 μM of Plerixafor. Interestingly, ^131^I-HZ262 uptake was highest in both U87-CXCR4 and H69 (high CXCR4 expression), whereas a structurally different ^76^Br-HZ270-1 was highest in the same cell lines, when compared to the other radiolabeled cyclam derivatives. ^76^Br-HZ270-1 and ^131^I-HZ262 achieved comparable high U87-CXCR4/U87-wt and H69/H82 uptake ratios: 2.96 ± 0.67 and 3.93 ± 0.74 for ^76^Br-HZ270-1, respectively, and 3.19 ± 1.02 and 3.81 ± 0.40 for ^131^I-HZ262, respectively. These promising results from *in vitro* studies supported proceeding to *in vivo* PET imaging of CXCR4 expression in tumor-bearing animal models, using the newly developed radiolabeled cyclam derivatives.

### CXCR4 Expression in Glioma and PCNSL Cell Lines and Tumors

**Fig. 3:**
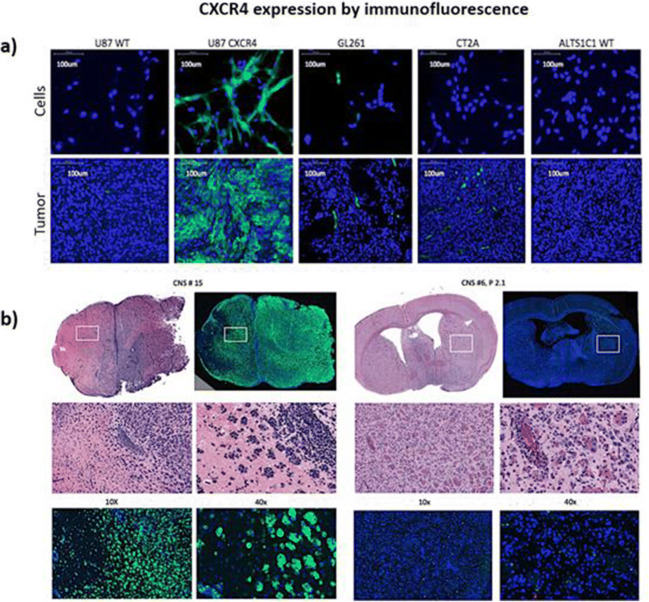
CXCR4 expression by immunofluorescence. Immunofluorescence staining for CXCR4 (green) and DAPI staining (blue) of tumor cell lines in culture (**a**), and two human primary CNS lymphomas (CNS #15 and CNS #6) growing in SCID mice (**b**), are shown. H&E staining of adjacent tissue sections are also shown (**b**).

A number of human and murine cell lines listed in Table 2 were evaluated for CXCR4 expression, and varying amounts of CXCR4 protein expression were observed in the different cell lines. Among all tested cell lines, the CXCR4 transduced U87 cells expressed the highest levels of CXCR4 protein (Fig. 3a). Both cytoplasmic and cell membrane localization of CXCR4 expression were seen by IF in CXCR4-transduced U87 cells, whereas little cytoplasmic and no cell membrane staining were seen in U87 WT cells (Fig. 1). We previously showed that ^68^Ga-Pentixafor uptake is high in U87-CXCR4 tumors, but not in U87 wild-type tumors [23], demonstrating the validity of the U87-CXCR4/U87-wt animal model. The PCNSL animal models also expressed varying amounts of CXCR4 (Table 2). The human PCNSL xenografts are highly infiltrative and cluster around blood vessels, with little (or no) associated brain edema apparent on H&E staining (Fig. 3b). Different glioma cell lines and PCNSL xenografts expressed different amounts of CXCR4 (green immunostain), providing a range of expression levels for testing the CXCR4 imaging efficacy of the radiolabeled cyclam derivatives in these animal models.

### Uptake of Halogenated Cyclam Derivatives in s.c. Glioma and Lung Cancer Tumors

**Fig. 4:**
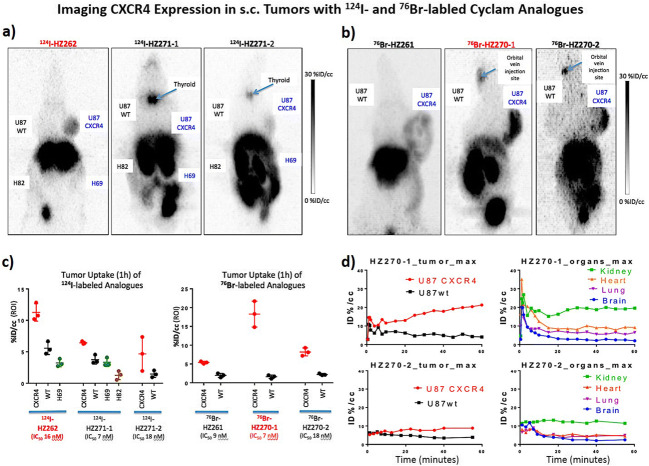
Imaging CXCR4 expression in s.c. tumors with ^124^I- and ^76^Br-labeled Cyclam analogues. MicroPet imaging of ^124^I-labeled HZ262, HZ271-1 and HZ271-2 (**a**). MicroPet imaging of ^76^Br-labeled HZ262, HZ270-1 and HZ270-2 (**b**). Quantitation of tumor radioactivity (%ID/cc) at 1 h post-i.v. injection (**c**). Time course of ^76^Br-HZ270-1 and ^76^Br-HZ270-2 uptake in U87 CXCR4-expressing and wild-type tumors and in different organs (**d**).

The imaging and uptake of 6 different radiolabeled cyclam derivatives were studied in subcutaneous tumor-bearing animals (animals bearing either 2 glioma or 2 glioma plus 2 lung cancer tumors) (Fig. 4). Consistent with the *in vitro* uptake results (Fig. 2), ^131^I-HZ262 and ^76^Br-HZ270-1 were accumulated highest in U87-CXCR4 s.c. tumors following i.v. injection (Fig. 3a–c). The kinetics of tumor accumulation and organ clearance of ^76^Br-HZ270-1 and ^76^Br-HZ270-2 is shown in Fig. 3d. Radioactivity continued to increase in the U87-CXCR4 tumors but was washed out from the U87 wt (non-CXCR4-exressing) control tumors, over 60 min. Normal brain had a rapid wash out profile with very low levels beyond 15 min, as radioactivity cleared from the blood (Fig. 4d). The imaging studies of the s.c. tumor models confirmed the advantage of ^76^Br-HZ270-1 for imaging CXCR4 expression, although high uptake was noted in the kidneys, intestine, and liver. Biodistribution studies confirmed the imaging studies. Comparing three radiolabeled cyclam derivatives, ^76^Br-HZ270-1 was accumulated most avidly by U87-CXCR4 tumors and had the lowest overall systemic organ levels at 24 h post i.v. injection (Table 3). Brain radioactivity was lowest of all organs sampled. Based on these studies, ^76^Br-HZ270-1 was selected for imaging intracranial CXCR4-expressing tumors.

**Table 3:** Biodistribution of ^124^I^−^ and ^76^Br-labeled cyclam analogues—24 h after i.v.

	^124^I-HZ271-1	^76^Br-HZ270-1	^76^Br-HZ270-2
	%ID/g (*n* = 3)	%ID/g (*n* = 3)	%ID/g (*n* = 3)
Tumors			
U87 CXCR4	2.0 ± 0.3	9.5 ± 1.3	4.8 ± 1.6
U87 WT	0.96 ± 0.21	1.6 ± 0.7	1.5 ± 0.2
H69	2.3 ± 0.2	–	–
H82	1.1 ± 1.0	–	–
Organs			
Blood	1.5 ± .05	0.59 ± 0.12	1.6 ± 0.5
Heart	2.0 ± 0.4	1.7 ± 0.3	2.3 ± 0.4
Lungs	2.3 ± 1.4		
Liver	30 ± 23	7.6 ± 1.3	15.3 ± 3.5
Spleen	1.3 ± 0.7	2.1 ± 0.8	2.5 ± 0.2
Kidneys	45.0 ± 5.7	41.3 ± 24.1	53.7 ± 12.6
Bone	0.99 ± 0.33	2.9 ± 0.5	1.9 ± 0.6
Brain	0.07 ± 0.04	0.14 ± 0.04	0.53 ± 0.01

### Imaging Intracranial CXCR4-Expressing Gliomas and PCNSL with ^76^Br-HZ270-1 and PET

**Fig. 5:**
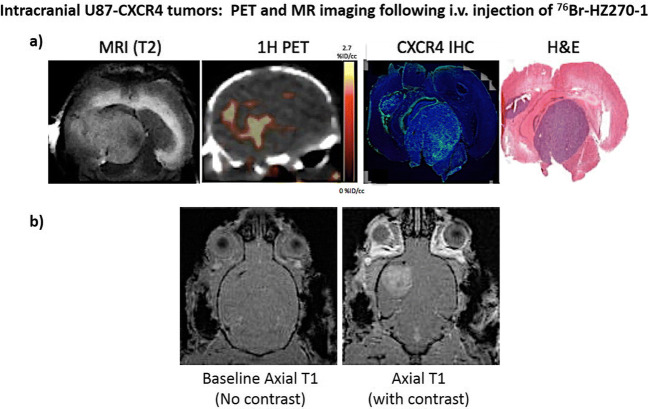
PET and MR imaging of an intracranial U87-CXCR4 tumor following i.v. injection of ^76^Br-HZ270-1. Image comparisons of the same intracerebral U87-CXCR4 glioma with approximate co-registration (**a**). Pre- and post-contrast MRI of an intracerebral U87-CXCR4 glioma (**b**)

**Fig. 6:**
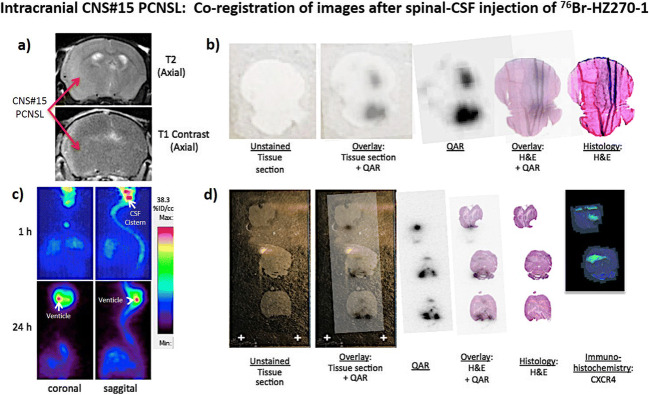
Co-registration of images after spinal-CSF injection of ^76^Br-HZ270-1: Intracranial CNS#15 PCNSL. A T2 and a post-contrast T1 MRI of an intracerebral CNS#15 PCNSL (**a**); confirmation tumor CXCR4 expression, as seen in Fig. 3b. Coronal and saggital PET images of ^76^Br-HZ270-1 radioactivity, 1 h and 24 h after spinal-CSF injection, in an animal bearing an intracerebral CNS#15 PCNSL (**b**). Co-registration of images after spinal-CSF injection of ^76^Br-HZ270-1 in an animal bearing an intracerebral CNS#15 PCNSL (**c**, **d**). The animals in **a** and **b** and in **c** and **d** are different.

Two high CXCR4-expressing intracranial tumors were selected (U87-CXCR4 and PCNSL-15; Table 2) for PET imaging with ^76^Br-HZ270-1. The initial study involved U87-CXCR4 intracranial xenografts and i.v. injection of ^76^Br-HZ270-1 (Fig. 5a). Very low levels of radioactivity were visualized in the PET scan, despite moderate CXCR4 expression of CXCR4 visualized on immunohistochemistry. Similar results were obtained with intracranial PCNSL-15 CXCR4-expressing tumors and i.v. injection of ^76^Br-HZ270-1 (not shown). MRI with and without contrast showed a heterogeneous modest breakdown of the blood-tumor barrier in U87-CXCR4 intracranial xenografts (Fig. 5b), whereas little or no breakdown of the blood-tumor barrier was observed in intracranial PCNSL-15 tumors (Fig. 6a).

To bypass the blood-tumor barrier in the intracranial PCNSL-15 tumor model, ^76^Br-HZ270-1 was injected directly into the spinal-CSF and PET scans were performed at 1 and 24 h post injection (Fig. 6). In this tumor model, ^76^Br-HZ270-1 tracked from the mid-thoracic/lumbar region (spinal-CSF injection site) up to the brain over 24 h (Fig. 6b). Although the CSF PET tracking studies appeared to be encouraging (Fig. 6b) and suggestive of tumor imaging autoradiographic study (Fig. 6c), this proved not to be the case. Additional autoradiographic studies with appropriate CXCR4 IHC as well as H&E histology confirmed that ^76^Br-HZ270-1 radioactivity tracked-to and was localized-in brain CSF cisterns and not localized to CXCR4-expressing tumor (Fig. 6d).

## Discussion

High levels of CXCR4/CXCL12 expression in glioblastomas [6–8] and in PCNSL [3–5] have been shown previously. Activation of the CXCR4/CXCL12 pathway is considered to be a major contributor to tumor invasion of surrounding tissues [8, 9, 11]. In GBMs, CXCR4 expression correlates with poorer patient prognosis [9] and with a better treatment response to AMD3100 (a clinically approved CXCR4 inhibitor) [10–12]. In PCNSL, there is little or no literature describing the association of high CXCR4 expression and prognosis.

In a transgenic murine model of GBM, the CXCL12-CXCR4 signaling pathway in neural progenitor tumor cells is activated under hypoxic conditions through an autocrine-positive feedback mechanism, which promotes survival and cell-cycle progression (14). In an orthotopic glioblastoma mouse model, hypoxia induces upregulation of CXCL12 leading to the recruitment of CXCR4-positive bone marrow derived monocytes that stimulate the formation of new tumor blood vessels—vasculogenesis (15–17), and hypoxia is a well-known inducer of VEGF expression, leading to the induction of angiogenesis and tumor progression (18). Combination therapy consisting of XRT and CXCR4 inhibition with AMD3100 decreased angiogenesis and increased vasculogenesis and abrogated tumor recurrence within the 100-day follow-up period [10]. A clinical case report describes a remarkable response in a patient with GBM [33]; this is the first reported GBM patient treated successfully with a CXCR4 inhibitor (Plerixafor/AMD3100, as part of an adjuvant therapeutic strategy); the patient showed no evidence of recurrent disease during 2 years of continuous treatment.

The potential to develop radiolabeled Plerixafor analogues for imaging CXCR4 expression in brain tumors exists and could be used for selecting patient targeted treatment and for monitoring treatment efficacy. The main focus of current study was to develop radiolabeled the derivatives of Plerixafor and AMD3465 that crosses the BBB and tumor cell membranes for PET imaging of CXCR4. A future objective, after validating a CXCR4 imaging paradigm, would be to screen brain tumor patients for anti-CXCR4 targeted-radiotherapy.

Established peptide-based radioligands are currently available for imaging CXCR4 expression, particularly ^68^Ga-pentixafor and ^68^Ga-NOTA-NFB [22, 23]. They have been shown to be very effective in systemic tumors, such as multiple myeloma [14]. However, imaging CXCR4 expression in brain tumors with ^68^Ga-pentixafor and ^68^Ga-NOTA-NFB [21, 22] has been more limited. There are at least two potential explanations for this limitation: (i) the blood-brain barrier (BBB) may restrict access of radiolabeled peptides to many CXCR4-expressing tumor cells and (ii) a considerable fraction of CXCR4 staining is intracellular and intracellular CXCR4 may not be accessible to the peptides.

FDA-approved AMD3100 (Plerixafor) for non-Hodgkin’s lymphoma and multiple myeloma (2008) has two cyclam that is suitable for complexation with radiometals, including ^64^Cu, ^67^Ga/^68^Ga, and ^99m^Tc [15] [16–18]. Even though ^64^Cu-complexed Plerixafor AMD3465 showed high tumor and liver uptake *in vivo*, the additional positive charge from radiometal might prevent it from crossing BBB and target CXCR4-positive gliomas. Since we previously tried to develop a lipophilic ^18^F-labeled **3** (AMD3465 analogue without a cyclam moiety), which had no affinity to CXCR4, we proposed to develop new derivatives of AMD3100 and AMD3465 *via* radiohalogenation on the phenyl moiety. Poty et al. reported that AMD3100 could be functionalized on the phenyl moiety with different linkers, such as ethylenediamine or diamino-polyethylene glycol for further coupling with chelators [34]. The design of new cyclam derivatives based on radiohalogenating the phenyl moiety directly is a novel strategy that has not been previously explored, to the best of our knowledge. We show that such modifications have no or minimal effect on binding to the CXCR4 target. The “cold” halogenation and radiosynthesis of the 6 cyclam derivatives was successfully obtained with the purity of greater than 98 %. Furthermore, the iodinated and brominated cyclam derivatives we developed had structure-based estimates of lipophilicity (LogP estimates) that were in a range that suggested rapid transfer across the BBB and tumor cell membranes.

The competitive binding assay showed the cyclam group was the most sensitive to maintain the affinity to CXCR4 [35, 36]. Both iodinated and brominated cyclam derivatives showed similar nanomolar affinity to CXCR4 receptor, which approved our assumption in general. If we look into the structure-activity relationship closely, the bromination on the phenyl moiety had less influence on binding than iodination in the same position. In addition, halogenation at the 3rd position of phenyl moiety of AMD3465 derivatives resulted in a slight better affinity than halogenation at the 2nd position of phenyl moiety, making the 3rd position a better choice for further ligand optimization. The cell uptake studies and the *in vivo* uptake studies (in the s.c. tumor models) were generally consistent with the results of *in vitro* CXCR4-binding studies. It is worth noting that there was no *in vivo* de-halogenation of the three radiobrominated ligands, and ^124^I-AMD3100 (a bicyclam) showed no *in vivo* de-iodination on PET imaging. However, ^124^I-labeled AMD3465 mono cyclam derivatives did show de-halogenation with significant ^124^I radioactivity localized to the thyroid. This suggests that the cyclam group is the sensitive group for maintaining affinity and *in vivo* stability, and it may significantly influence the accumulation of ligand in CXCR4-positive tumors.

Although we did not evaluate the *in vitro* and *in vivo* stability of all tested ligands, the PET images in Fig. 4 show very different patterns of ^124^I accumulation in the thyroid, indicating a significant decomposition *in vivo* of ^124^I-HZ271-1 compared to ^124^I-HZ262. The differences in biodistribution and deiodination *in vivo* are likely to be factors (in addition to the binding affinity) that determine ligand accumulation in CXCR4-positive tumors. Even though ^124^I-HZ262 has a lower binding affinity than ^124^I-HZ271-1, the greater *in vivo* stability and more favorable biodistribution appear to contribute to the greater accumulation of ^124^I-HZ262 in CXCR4-positive tumors.

We identified ^76^Br-HZ270-1 as the best of the 6 radioligands for imaging CXCR4 expression in the s.c. tumor models. Then, we tested ^76^Br-HZ270-1 in appropriate murine orthotopic models of GBM and PCNSL to validate the imaging efficacy of this radiolabeled cyclam derivative two brain tumor models that express high levels of CXCR4 protein. However, ^76^Br-HZ2701 was unable to successfully image a CXCR4-expressing intracranial glioma (U87-CXCR4) or a CXCR4-expressing intracranial PCNSL (PCNSL-15) following i.v. or spinal-CSF injection. Two reasons for this result are apparent: (i) the radioligand (^76^Br-HZ270-1) was actually hydrophilic (LogD = − 0.72), not lipophilic as estimated from a structure-based calculation (ChemDraw/Chem3D/ChemFinder Software, PerkinElmer Informatics) (estimated LogP = 2.17); (ii) both the intracranial U87-CXCR4 and PCNSL-15 animal tumor models had comparatively intact BBB and BTB, as seen on contrast MRI.

It should be noted that AMD3100 and AMD3465 are highly protonated (6 and 4 protons, respectively; Fig. 1a) and are very basic in aqueous solution. Similarly, the 6 radiolabeled cyclam derivatives are also highly protonated (Fig. 1a). Compared to bromine, the larger hydodynamic diameter of iodine is able to bring hydrophilic AMD3465 into a more hydrophobic profile; however, the poor *in vivo* stability and low accumulation in s.c. xenografts prevented further evaluation in glioma models. Moving forward, the challenge will be to increase the lipophilicity of radiolabeled cyclam derivatives (*e.g.*, by reducing the number of protons to 2 or 1) without losing the high affinity to CXCR4 (low nM range). If successful, these novel agents could be used to screen/select of chemotherapy-resistant patients with recurrent GBM or PCNSL for targeted anti-CXCR4 chemotherapy (singly or in combination with other treatments) in the future. Furthermore, if successful, it would be possible to develop a theranostic strategy, incorporating both imaging for patient selection and targeted radiotherapy for treatment, using ^76/77^Br-labeld ligands in the future.

## Conclusions

We successfully designed and synthesized new derivatives of AMD3100 and AMD3465 based on radiohalogenating the phenyl moiety directly, with greater than 98 % purity. Such modifications have no or minimal effect on binding to the CXCR4 target and suggest that the structure of the cyclam moiety is most important for specific CXCR4 binding. PET imaging of CXCR4 expression with radiolabeled halogenated derivatives was successful in s.c. located tumors, but not in CNS located tumors. This was largely due to the following: (i) the hydrophilicity of the radiolabeled analogues—as reflected in the “measured” the radiotracer distribution (LogD) in octanol/PBS—which stands in contrast to the structure-based estimate of LogP, which was the rationale for initiating the study; (ii) the presence of a modest BTB in intracranial U87-CXCR4 gliomas and an intact BBB/BTB in the intracranial PCNSL animal model.

### Question

Would halogenated cyclam derivatives, thought to be hydrophobic on structure-based estimates of their lipophilicity, provide new radiolabeled ligands for imaging CXCR4 expression in gliomas and PCNSL?

### Pertinent Findings

Six iodinated and brominated cyclam derivatives with high affinity (low nM range) for CXCR4 were successfully synthesized and tested *in vitro* and *in vivo,* using appropriate cell lines and murine models of GBM and PCNSL*.* PET imaging of CXCR4 expression in s.c. tumors was successful, but due to the hydrophilicity of the radiolabeled cyclam analogues and the presence of an intact BTB in the animal models, intracranial tumor visualization was poor.

### Implications for Patient Care

CXCR4 is one of several “chemokine” receptors expressed on malignant tumors (including GBM and PCNSL). Although ^68^Ga-pentixafor and ^68^Ga-NOTA-NFB have been shown to effectively image CXCR4 expression in myeloma and other systemic malignancies, imaging CXCR4 expression in brain tumors with these ligands has been more limited. The rationale for our studies was that cyclam derivates labeled with iodine or bromine would more effectively cross the blood-brain barrier and enable imaging of CXCR4, in contrast to peptides and radiometal complexes. Since a structurally similar anti-CXCR4 drug (AMD3100) is FDA-approved, this would facilitate clinical translation of these radiolabeled molecules for imaging CXCR4 and potentially be extended to targeted radiotherapy.

## Electronic Supplementary Material

ESM 1 (DOCX 4132 kb)
