# Supplementary material for: Imaging CXCR4 Expression with Iodinated and Brominated Cyclam Derivatives
Source: Mol Imaging Biol. 2020 Apr 1;22(5):1184–96. doi: 10.1007/s11307-020-01480-1 (PMC7497443; doi:10.1007/s11307-020-01480-1)
Supplement: Supplementary file 1 — (DOCX 4132 kb) [file 11307_2020_1480_MOESM1_ESM.docx]

Title: Imaging CXCR4 expression with Iodinated and Brominated Cyclam Derivatives

Authors: Hanwen Zhang*^,1^, Masatomo Maeda*^,2,5^, Masahiro Shindo^2.5^ , Myat Ko^2^, Mayuresh Mane^2^, Christian Grommes^2^, Wolfgang Weber^1,4^, Ronald Blasberg^1,2,3,†^

Affiliations: ^1^ Department of Radiology, ^2^ Department of Neurology, ^3^ Molecular Pharmacology Program, Memorial Sloan Kettering Cancer Center, ^4^ Department of Nuclear Medicine, Technical University Munich, Germany, ^5^ Department of Neurosurgery, Nozaki Tokushukai Hospital, Osaka, Japan

* Contributed equally to the manuscript

^†^ Corresponding author: Ronald G. Blasberg, M.D.

Departments of Neurology and Radiology, MH (Box 52)

Molecular Pharmacology & Chemistry Program, SKI

Memorial Sloan Kettering Cancer Center (MSKCC)

Zuckerman Research Center (ZRC), Z-2060

1275 York Avenue

New York, N.Y.   10065

Telephone:  (646) 888-2211

e-mail:     [blasberg@neuro1.mskcc.org](mailto:blasberg@neuro1.mskcc.org)

Supplemental Figure 1: Representative ligand binding assays targeting CXCR4 expression on U87-CXCR4 cells.

Supplemental Figure 2: Precursors identified with high resolution mass spectroscopy.

**HZ262pro**

**
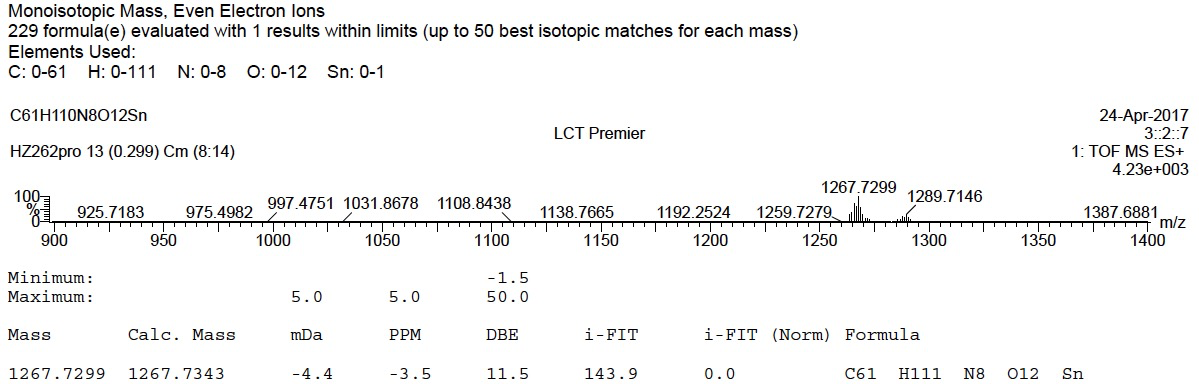
**

**HZ271pro-1
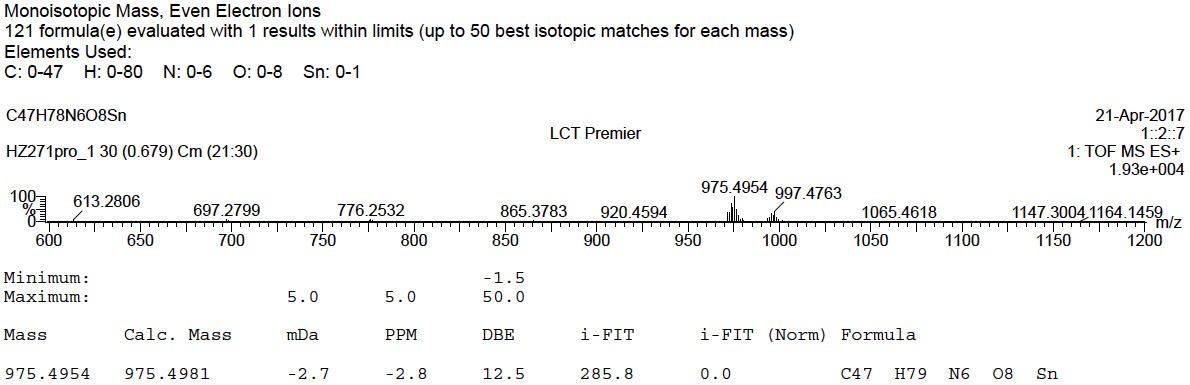
**

**HZ271pro-2**

**
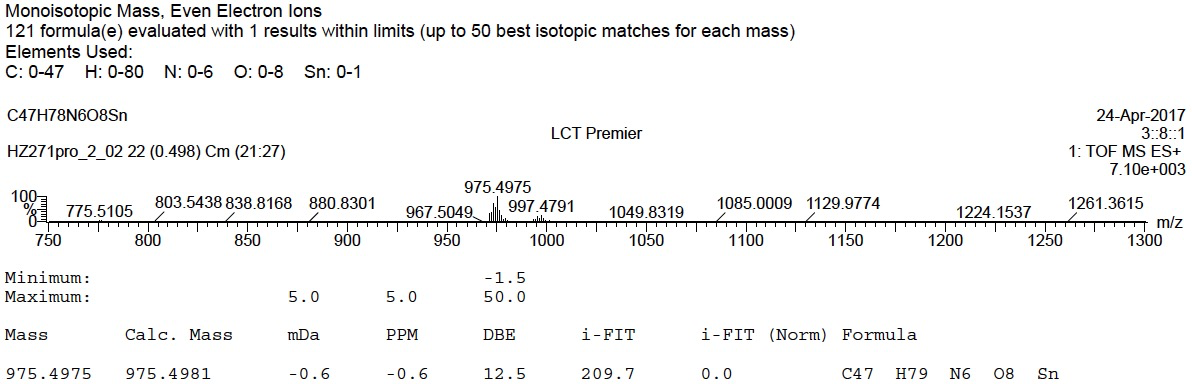
**
